# Secondary Infections with Ebola Virus in Rural Communities, Liberia and Guinea, 2014–2015

**DOI:** 10.3201/eid2209.160416

**Published:** 2016-09

**Authors:** Kim A. Lindblade, Tolbert Nyenswah, Sakoba Keita, Boubakar Diallo, Francis Kateh, Aurora Amoah, Thomas K. Nagbe, Pratima Raghunathan, John C. Neatherlin, Mike Kinzer, Satish K. Pillai, Kathleen R. Attfield, Rana Hajjeh, Emmanuel Dweh, John Painter, Danielle T. Barradas, Seymour G. Williams, David J. Blackley, Hannah L. Kirking, Monita R. Patel, Monica Dea, Mehran S. Massoudi, Albert E. Barskey, Shauna L. Mettee Zarecki, Moses Fomba, Steven Grube, Lisa Belcher, Laura N. Broyles, T. Nikki Maxwell, Jose E. Hagan, Kristin Yeoman, Matthew Westercamp, Joshua Mott, Frank Mahoney, Laurence Slutsker, Kevin M. DeCock, Barbara Marston, Benjamin Dahl

**Affiliations:** Centers for Disease Control and Prevention, Atlanta, Georgia, USA (K.A. Lindblade, P. Raghunathan, J.C. Neatherlin, M. Kinzer, S.K. Pillai, K.R. Attfield, R. Hajjeh, J. Painter, D.T. Barradas, S.G. Williams, D.J. Blackley, H.L. Kirking, M.R. Patel, M. Dea, M.S. Massoudi, A.E. Barskey, S.L. Mettee Zarecki, S. Grube, L. Belcher, L.N. Broyles, T.N. Maxwell, J.E. Hagan, K. Yeoman, M. Westercamp, J. Mott, F. Mahoney, L. Slutsker, K.M. DeCock, B. Marston, B. Dahl);; Liberia Ministry of Health and Social Welfare, Monrovia, Liberia (T. Nyenswah, F. Kateh, T.K. Nagbe, E. Dweh, M. Fomba);; Guinea Ministry of Health and Public Hygiene, Conakry, Guinea (S. Keita); World Health Organization, Geneva, Switzerland (B. Diallo);; New York City Department of Health and Mental Hygiene, New York, New York, USA (A. Amoah)

**Keywords:** Ebola virus, viruses, Ebola virus disease, hemorrhagic fever, disease outbreaks, secondary infections, hospitalization, public health, quarantine, rural communities, patient isolation, Liberia, Guinea, western Africa

## Abstract

Persons who died of Ebola virus disease at home in rural communities in Liberia and Guinea resulted in more secondary infections than persons admitted to Ebola treatment units. Intensified monitoring of contacts of persons who died of this disease in the community is an evidence-based approach to reduce virus transmission in rural communities.

Transmission of Ebola virus occurs through direct contact with blood or other body fluids of an infected person after symptoms have developed. During an outbreak of Ebola virus disease (EVD), monitoring persons (termed contacts) who have exposure to persons with EVD is the most effective way to identify and isolate new cases rapidly before transmission can occur (*1*). At the height of the 2014–2015 epidemic in West Africa, response teams were monitoring daily >7,000 contacts in Liberia, 8,900 in Sierra Leone, and 2,800 in Guinea (Emergency Operations Center, Centers for Disease Control and Prevention, Atlanta, GA, USA, pers. comm.).

The World Health Organization (WHO) guidelines for monitoring contacts of -persons with EVD treat all contacts equally (*1*). However, when resources are limited, evidence-based criteria for identifying cases of EVD most likely to result in secondary infections could help to optimize control. We analyzed data from outbreaks in rural areas of Liberia and Guinea to determine whether intensifying monitoring of contacts of persons with EVD who died at home in the community was warranted.

## The Study

Under the leadership of the Ministries of Health (MOHs) of Liberia (July–December 2014) (*2*) and Guinea (December 2014–June 2015), epidemiologists from multiple agencies investigated rural outbreaks of EVD. Within a community outbreak, field epidemiologists identified case-patients, monitored their contacts, and developed diagrams of infection sequences on the basis of interviews with patients, families, and community members. Transmission diagrams began with the first case identified in the community (index case) and ended when all known contacts had completed 21 days of monitoring with no new cases identified. In both countries, information from EVD case report forms included age, sex, date of symptom onset, date of isolation in an Ebola treatment unit (ETU), and date of recovery or death. Data from Guinea also included whether the person who died at home received a safe and dignified burial performed by trained teams (*3*). These investigations were conducted as part of the Ebola public health response in West Africa and were not considered to be human subjects research.

Data for Liberia and Guinea were combined. We used generalized estimating equations with a negative binomial distribution to compare the number of secondary infections between groups, including between persons with EVD who died at home in the community and those admitted to an ETU, between persons who were severely ill (death <3 days after admission) and those less ill (death >3 days of admission or recovery) at the time of admission to an ETU, and between persons who were buried safely by trained burial teams and those buried by untrained persons (in Guinea only). Additional details on statistical analyses are included in the Technical Appendix.

Data were available for 347 persons with EVD from 17 transmission chains; 240 (69%) persons were confirmed by using laboratory analysis (real-time PCR) as having EVD (Table). Most (185, 53%) persons with EVD were admitted to an ETU, of whom 102 (55%) died, 78 (42%) recovered, and 5 (3%) had a missing outcome. A total of 162 (47%) persons were not admitted to an ETU, of whom 157 (97%) died at home in the community and 4 (2%) recovered without hospitalization (3 had confirmed cases and 1 had a probable case). The overall case-fatality rate was 76% (95% CI 71%–81%).

**Table:** Characteristics of persons with Ebola virus disease in rural areas of Liberia and Guinea, 2014–June 2015*

Characteristic	Liberia, n = 165	Guinea, n = 182	Total, n = 347
No. transmission chains	9	8	17
Laboratory-confirmed EVD	114 (69)	126 (69)	240 (69)
Outcome			
Admitted to an ETU and recovered	49 (30)	29 (16)	78 (22)
Admitted to an ETU and died	51 (31)	51 (28)	102 (29)
Admitted to an ETU and unknown outcome	0 (0)	5 (3)	5 (1%)
Died at home in the community	60 (37)	97 (53)	157 (45)
Recovered in the community	4 (2)	0 (0)	4 (1)
Generated ≥1 secondary EVD infections	37 (24)	62 (39)	99 (31)
Generated ≥1 secondary EVD infections by origin of source case			
Source case died in the community	31 (55)	51 (55)	82 (55)
Source case was admitted to an ETU	5 (5)	11 (16)	16 (10)
Source case survived in the community	1 (25)	0 (0)	1 (25)
No. days at risk for transmitting secondary infections in the community	5.8 (5.2–6.5)	8.1 (1.8–14.4)	6.8 (4.0–9.6)
Timing of death within an ETU			
Died <3 d after admission	12 (12)	12 (16)	24 (12)
Died ≥3 d after admission or recovered	85 (88)	65 (84)	150 (86)
Burial status of those who died at home in the community			
Safely buried	NA	38 (40)	NA
Not safely buried	NA	56 (60)	NA

*Values are no. (%) or no. (95% CI). Percentages are proportions of data not missing. ETU, Ebola treatment unit; EVD, Ebola virus disease; NA, not available.

**Figure 1:**
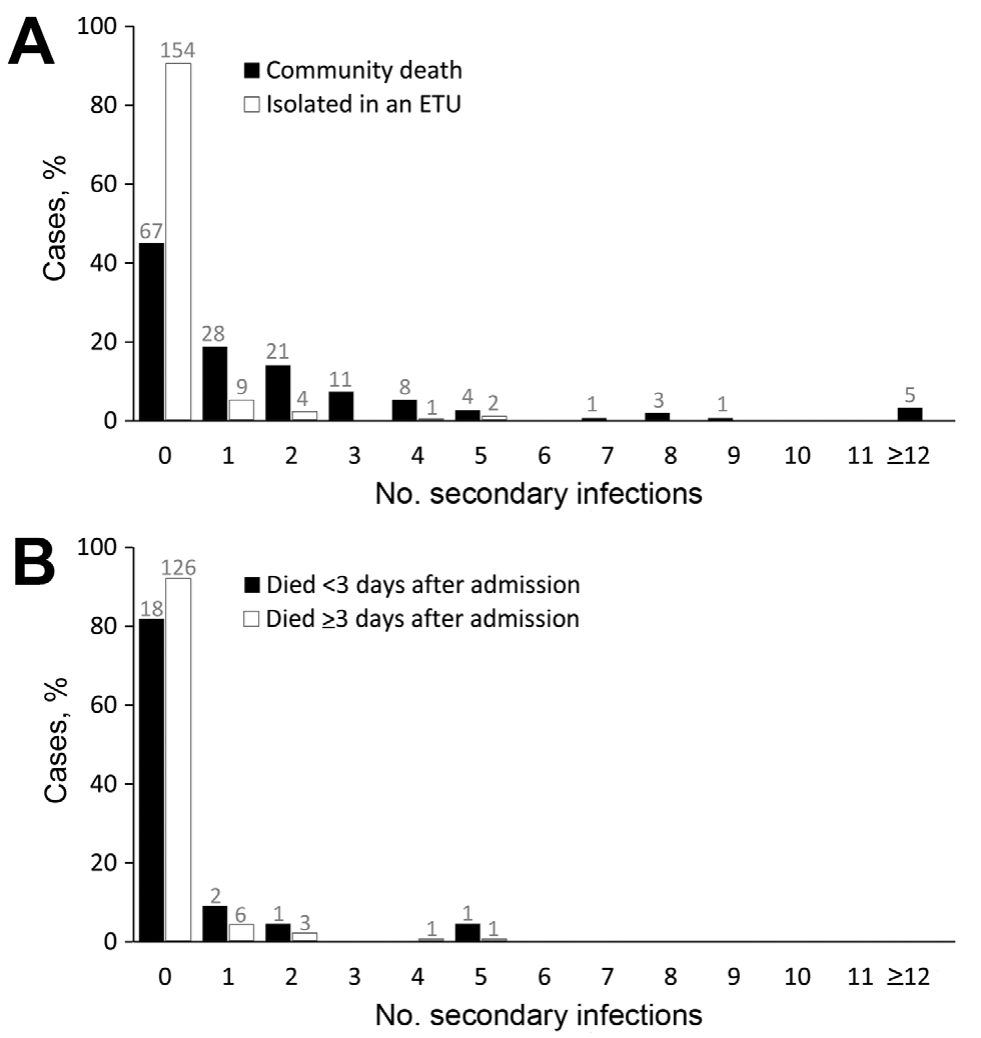
Percentile distribution, by number of secondary infections, of persons with Ebola virus disease (EVD) in rural outbreaks in Liberia and Guinea, 2014–2015. A) Comparison of persons with EVD who died at home in the community and those who were isolated and treated in Ebola treatment units (ETUs). B) Comparison of persons admitted to ETUs who died <3 days or ≥3 days after admission. Numbers above bars indicate actual counts.

We determined the number of secondary infections for 317 (91%) persons with EVD who had outcome data; 99 (31%) resulted in >1 secondary infections, and there were differences by outcome status (Table). When we excluded the 4 case-patients who recovered in the community without hospitalization, the mean number of secondary infections was significantly higher for persons with EVD who died at home in the community (1.8, 95% CI 1.3–2.3) than for persons admitted to an ETU (0.2, 95% CI 0.1–0.3; p = 0.003) (Figure 1, panel A).

**Figure 2:**
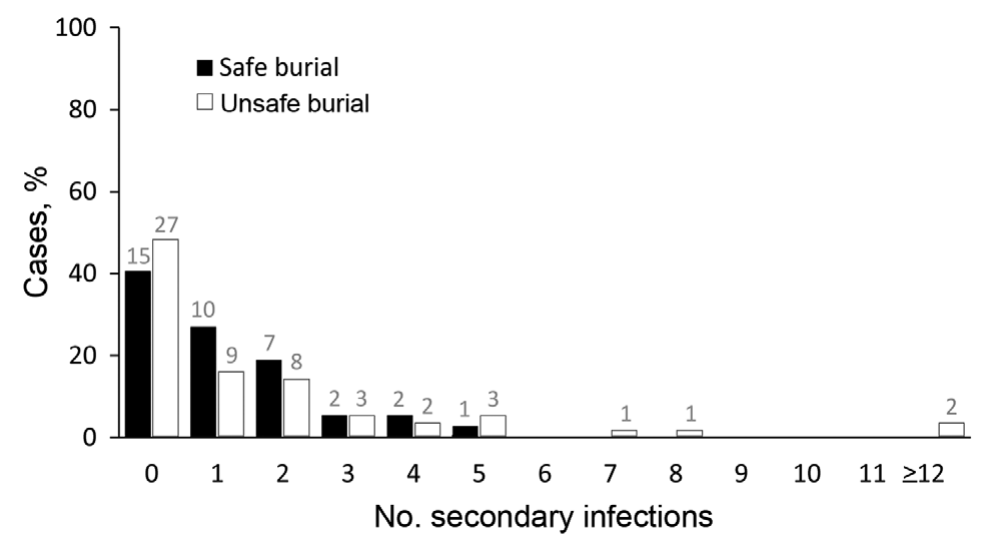
Percentile distribution, by number of secondary infections, of persons with Ebola virus disease in rural outbreaks who died at home in the community, by safe burial status, Guinea, 2015. Numbers above bars indicate actual counts.

We found no significant difference in the mean number of secondary infections between those who died <3 days after admission to the ETU (0.4, 95% CI 0.05–0.90) and those who died later in their hospitalization or recovered (0.1, 95% CI 0.06–0.30; p = 0.24) (Figure 1, panel B). We also found no significant difference in the mean number of secondary infections associated with cases of EVD in persons who received a safe burial (1.2, 95% CI 0.8–1.7) versus those who did not receive a safe burial (1.8, 95% CI 1.1–2.5; p = 0.40) (Figure 2).

## Conclusions

In rural outbreaks in Liberia and Guinea, Ebola virus transmission was driven by contact with persons who died of EVD at home, and isolation before death was associated with 88% fewer secondary infections. Possible reasons for a larger number of secondary cases associated with deaths at home in the community include 1) a higher per-contact probability of transmission caused by higher levels of viremia or more exposure to body fluids during terminal illness and death (*4*); 2) a greater number of contacts between uninfected persons and persons with EVD during their terminal illness or after death; and 3) a greater number of uninfected persons having contact with persons with EVD during their terminal illness or after death.

Because we did not find an increase in secondary infections according to severity of illness at the time of ETU admission (a proxy for level of viremia), we believe that factors associated with the death are critical in transmission. Traditional burial practices in West Africa include touching and washing the body after death (*5*). Extensive postmortem exposures to body fluids and skin could occur during that time, and postmortem studies of nonhuman primates have shown that Ebola virus is stable in body fluids for as long as 3 weeks (*6*). However, it is also likely that there is an increased number of secondary infections because more persons touch the corpse while paying respect to the deceased than would touch a living patient during their illness (*7*).

Yamin et al. used a stochastic modeling approach to integrate epidemiologic data on Ebola from Liberia to identify potential intervention targets (*8*). Similar to our data, they found that secondary cases were most associated with nonsurvivors, and that isolation within 4 days of symptom onset could eliminate disease transmission. Our findings differ slightly from those of Yamin et al. because they suggest that isolation of cases of EVD at any time before death would reduce transmission.

In Guinea, we did not find burials reported as safe to have had an effect on reducing the number of secondary infections. Although some of the safe burials might have been misclassified, it is more likely that traditional mourning practices occurred before safe burial teams arrived (*7*). Response to future outbreaks should emphasize prevention of exposure to Ebola virus during mourning and burial, and cadavers should be classified as safely buried only if they have not been touched after death.

Classifying contacts of persons who died of EVD at home in the community as high-risk, regardless of whether they were reported to have received a safe burial, is an evidence-based approach to prioritizing those persons who should receive more rigorous monitoring. Intensive follow-up could include assignment of highly trained staff to evaluate high-risk contacts more frequently, provision of incentives to complete the 21-day monitoring period, and housing high-risk persons in managed voluntary quarantine facilities.

Technical Appendix Additional information on statistical analysis for secondary infections with Ebola virus in rural communities, Liberia and Guinea, 2014–2015.

## References

[1] World Health Organization and Centers for Disease Control and Prevention. Implementation and management of contact tracing for Ebola virus disease. Geneva: The Organization; 2015.

[2] Lindblade KA, Kateh F, Nagbe TK, Neatherlin JC, Pillai SK, Attfield KR, Decreased Ebola transmission after rapid response to outbreaks in remote areas, Liberia, 2014. Emerg Infect Dis. 2015;21:1800–7 . 10.3201/eid2110.150912 26402477 PMC4593457

[3] World Health Organization. Field situation: how to conduct safe and dignified burial of a patient who has died from suspected or confirmed Ebola virus disease. Geneva: The Organization; 2014.

[4] Towner JS, Rollin PE, Bausch DG, Sanchez A, Crary SM, Vincent M, Rapid diagnosis of Ebola hemorrhagic fever by reverse transcription-PCR in an outbreak setting and assessment of patient viral load as a predictor of outcome. J Virol. 2004;78:4330–41 . 10.1128/JVI.78.8.4330-4341.2004 15047846 PMC374287

[5] Fairhead J. The significance of death, funerals and the after-life in Ebola-hit Sierra Leone, Guinea and Liberia: anthropological insights into infection and social resistance. Sussex (UK): Institute of Development Studies, University of Sussex; 2014.

[6] Prescott J, Bushmaker T, Fischer R, Miazgowicz K, Judson S, Munster VJ. Postmortem stability of Ebola virus. Emerg Infect Dis. 2015;21:856–9 . 10.3201/eid2105.150041 25897646 PMC4412251

[7] Victory KR, Coronado F, Ifono SO, Soropogui T, Dahl BA; Centers for Disease Control and Prevention. Ebola transmission linked to a single traditional funeral ceremony— Kissidougou, Guinea, December, 2014–January 2015. MMWR Morb Mortal Wkly Rep. 2015;64:386–8. 25879897 PMC5779538

[8] Yamin D, Gertler S, Ndeffo-Mbah ML, Skrip LA, Fallah M, Nyenswah TG, Effect of Ebola progression on transmission and control in Liberia. Ann Intern Med. 2015;162:11–7 . 10.7326/M14-2255 25347321 PMC4402942

