# Supplementary material for: Secondary Infections with Ebola Virus in Rural Communities, Liberia and Guinea, 2014–2015
Source: Emerg Infect Dis. 2016 Sep;22(9):1653–5. doi: 10.3201/eid2209.160416 (PMC4994349; doi:10.3201/eid2209.160416)
Supplement: Technical Appendix — Additional information on statistical analysis for secondary infections with Ebola virus in rural communities, Liberia and Guinea, 2014–2015. [file 16-0416-Techapp-s1.pdf]

Article DOI: http://dx.doi.org/10.3201/eid2209.160416

Secondary Infections with Ebola Virus in Rural Communities, Liberia and Guinea, 2014–2015

Technical Appendix

Generalized estimating equations with a log link and a negative binomial distribution were used to compare the number of secondary infections between 2 groups because a negative binomial distribution fit the observed count of the number of persons with Ebola virus disease by the number of secondary infections they generated (Pearson goodness of fit test statistic 7.85, degrees of freedom 7, p = 0.34) (*1*). An offset term of the log number of days at risk for transmission in the community (from symptom onset to death or admission to an Ebola treatment unit) was included in the model. Country of origin was included in models if found to be a confounder, and interactions between country and predictors were evaluated and reported if found to be statistically significant. An exchangeable correlation matrix was used to adjust the variance for clustering by transmission chain. We calculated 95% CIs for the mean number of secondary cases by group using bootstrapping with 2,000 resamples.

Reference

1. Data analysis examples SAS. Negative binomial regression. Los Angeles: UCLA Statistical Consulting Group [cited 2016 Jan 22]. http://www.ats.ucla.edu/stat/sas/dae/nbreg.htm/

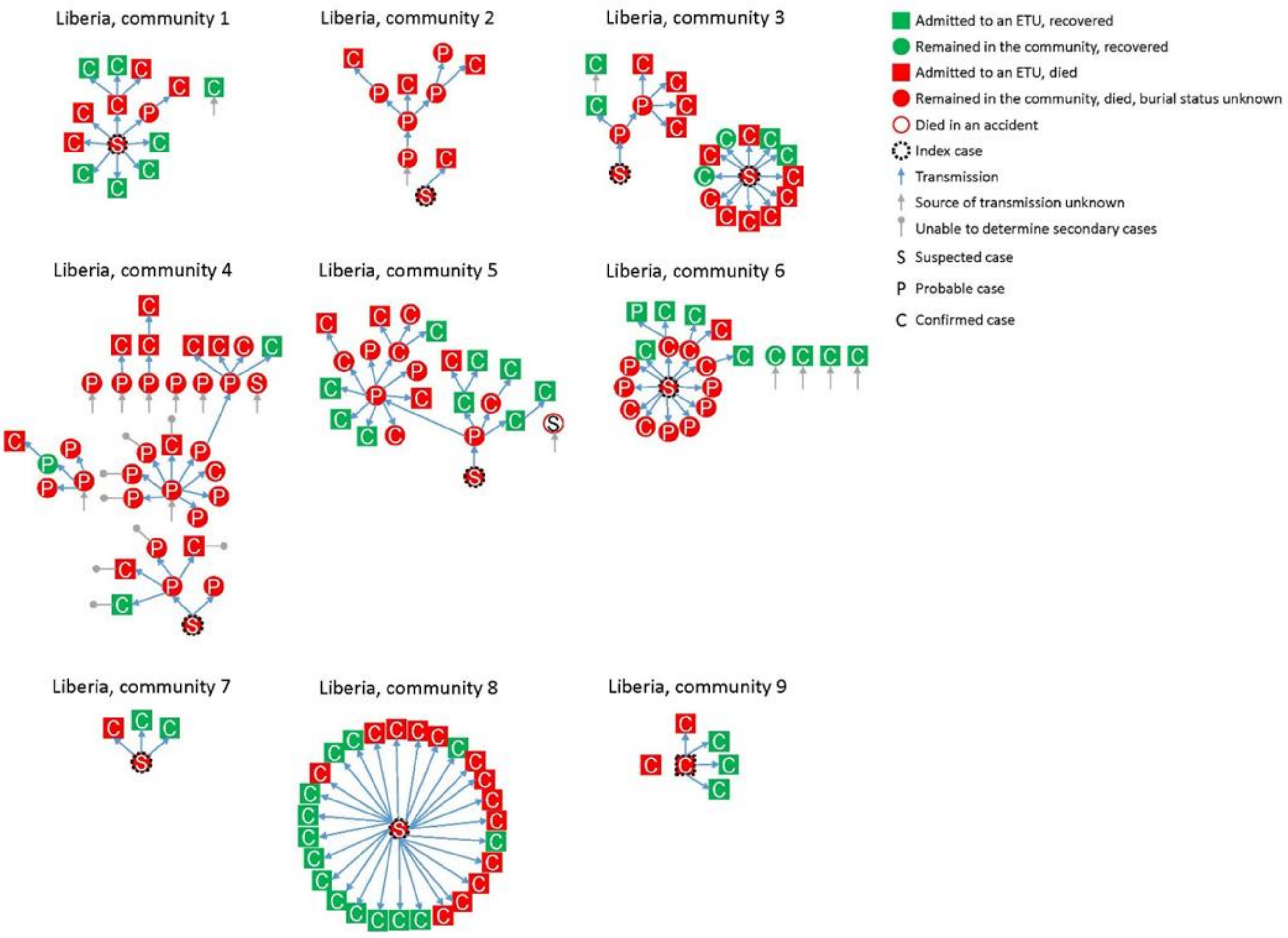

Technical Appendix Figure 1. Transmission diagrams for outbreaks of Ebola virus disease in 9 communities (1–9) in Liberia, 2014–2015. ETU, Ebola treatment unit.

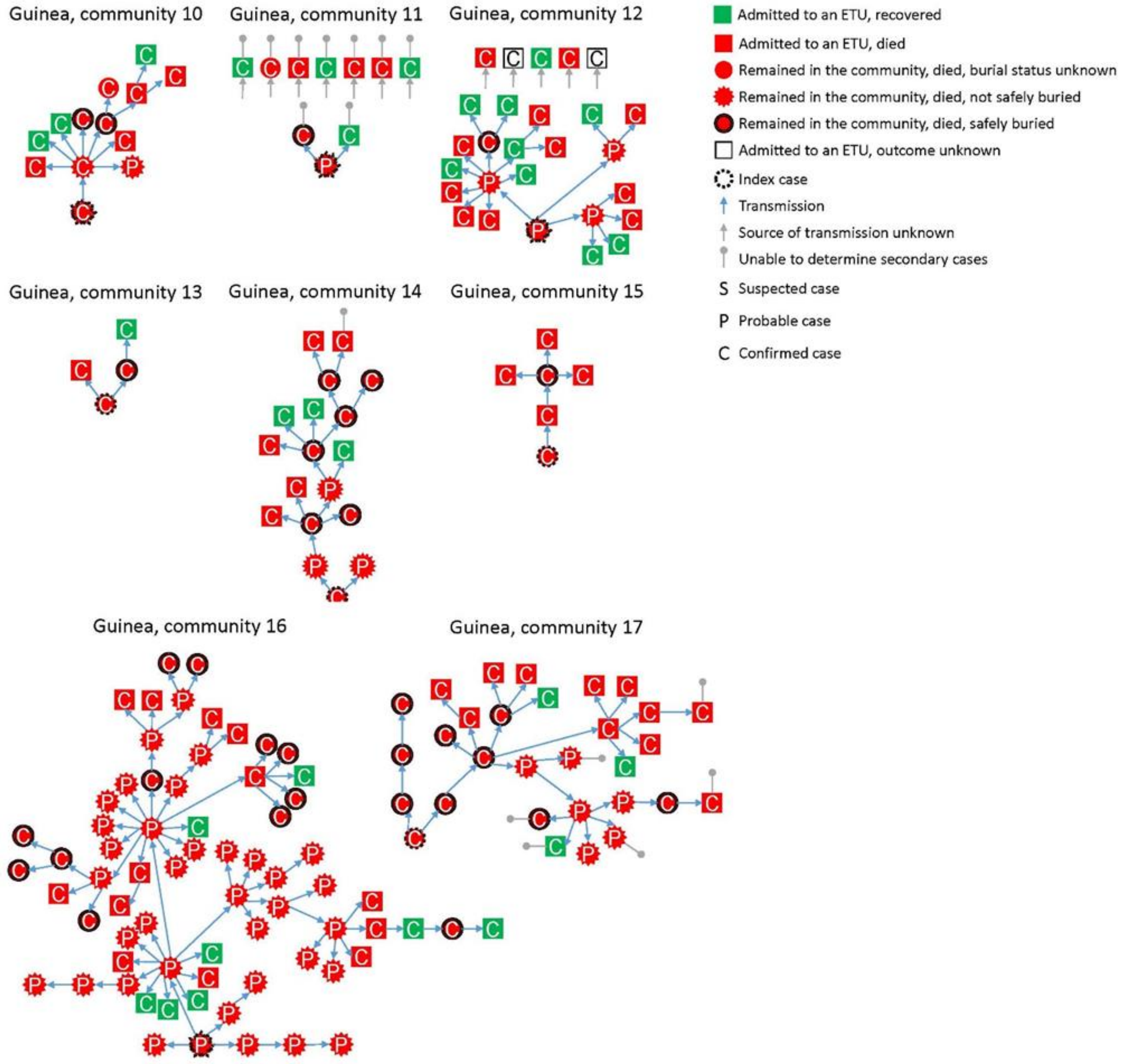

Technical Appendix Figure 2. Transmission diagrams for outbreaks of Ebola virus disease in 8 communities (10–17) in Guinea, 2014–2015. ETU, Ebola treatment unit.
